# New strategies to inhibit KEAP1 and the Cul3-based E3 ubiquitin ligases

**DOI:** 10.1042/BST20130215

**Published:** 2014-01-23

**Authors:** Peter Canning, Alex N. Bullock

**Affiliations:** *Structural Genomics Consortium, University of Oxford, Old Road Campus, Roosevelt Drive, Oxford OX3 7DQ, U.K.

**Keywords:** antioxidant response, cancer, cell signalling, degradation, drug design, ubiquitylation, ARE, antioxidant-response element, BACK, BTB and C-terminal Kelch, BTB, broad complex/tramtrack/bric-à-brac, CRL, Cullin–RING ligase, HECT, homologous with E6-associated protein C-terminus, KEAP1, Kelch-like ECH-associated protein 1, KLHL, Kelch-like, MATH, meprin and TRAF (tumour-necrosis-factor-receptor-associated factor) homology, Nrf2, nuclear factor erythroid 2-related factor 2, POZ, pox virus and zinc finger, Rbx, RING box protein, RING, really interesting new gene, SPOP, speckle-type POZ protein

## Abstract

E3 ubiquitin ligases that direct substrate proteins to the ubiquitin–proteasome system are promising, though largely unexplored drug targets both because of their function and their remarkable specificity. CRLs [Cullin–RING (really interesting new gene) ligases] are the largest group of E3 ligases and function as modular multisubunit complexes constructed around a Cullin-family scaffold protein. The Cul3-based CRLs uniquely assemble with BTB (broad complex/tramtrack/bric-à-brac) proteins that also homodimerize and perform the role of both the Cullin adapter and the substrate-recognition component of the E3. The most prominent member is the BTB–BACK (BTB and C-terminal Kelch)–Kelch protein KEAP1 (Kelch-like ECH-associated protein 1), a master regulator of the oxidative stress response and a potential drug target for common conditions such as diabetes, Alzheimer's disease and Parkinson's disease. Structural characterization of BTB–Cul3 complexes has revealed a number of critical assembly mechanisms, including the binding of an N-terminal Cullin extension to a bihelical ‘3-box’ at the C-terminus of the BTB domain. Improved understanding of the structure of these complexes should contribute significantly to the effort to develop novel therapeutics targeted to CRL3-regulated pathways.

## Cullin–RING ligases

Specific patterns of mono- or poly-ubiquitylation are used by the cell to control protein function or stability. These common post-translational modifications involve a three-enzyme cascade that directs the covalent linkage of the small protein ubiquitin on to a target protein lysine residue. An E1 ubiquitin-activating enzyme uses ATP to activate the ubiquitin for linkage to an E2 ubiquitin-conjugating enzyme. The E2–ubiquitin associates with an E3 ubiquitin ligase, which immobilizes and orients a specific substrate ready for ubiquitin conjugation [1]. E3 ligases are best known for their recruitment of substrates for degradation by the ubiquitin–proteasome system. They possess extraordinary specificity for a vast array of substrates and, as such, are considered promising, if challenging, targets for drug discovery [2]. E3 ligases may be divided into two major classes, HECT (homologous with E6-associated protein C-terminus) or RING (really interesting new gene) type, depending on whether they contain a HECT or a RING domain [3]. Ubiquitylation by HECT class E3s proceeds via an E3–ubiquitin intermediate, whereas RING class E3s conjugate ubiquitin directly to the substrate.

The multisubunit CRLs (Cullin–RING ligases) represent the largest class of E3 ligase. CRLs are constructed around a Cullin family protein (Cul1–Cul5 or Cul7) that forms an extended scaffold for protein interaction [4]. Specific substrate receptor proteins assemble with the Cullin N-terminal domain, typically via an adapter protein, whereas the globular C-terminal domain binds a RING box protein (Rbx1 or Rbx2). The RING domain recruits the activated E2–ubiquitin conjugate in advance of ubiquitin transfer. Structural data have been invaluable in detailing many aspects of CRL function. The structure of the Cul1-based SCF (Skp1–Cul1–F-box) complex defined the prototypical CRL architecture [5]. In this example, Skp1 serves as the adapter protein and Skp2 as the F-box-containing substrate receptor. Crystal structures have also defined how Cullin NEDDylation enhances the association of the E2–ubiquitin and substrate [6] and how this ubiquitylation is inhibited by CAND1 (Cullin-associated and NEDDylation-dissociated 1) [7].

## Cullin-3-based CRLs employ BTB domain proteins as substrate-specific adapters

Cullin-3-based CRLs recruit BTB (broad complex/tramtrack/bric-à-brac) domain proteins as their substrate-specific adapters. The BTB domain, or POZ (pox virus and zinc finger) domain is a protein–protein interaction domain that was first characterized by the crystal structure of the PLZF (promyelocytic leukaemia zinc finger protein) [8] and shares a conserved fold with both Skp1 and the Cul2/5 adapter Elongin C. Unusually, BTB proteins also contain an additional protein–protein interaction domain, such as a MATH [meprin and TRAF (tumour-necrosis-factor-receptor-associated factor) homology], ZnF (zinc finger) or Kelch domain, to function as both the adapter and substrate receptor module [9]. Furthermore, the BTB domain typically folds as a homodimer, leading to CRL dimerization.

The KLHL (Kelch-like) family of proteins represent the largest group of BTB-containing substrate receptors. These are characterized by an N-terminal BTB domain, a C-terminal Kelch domain and an intervening BACK (BTB and C-terminal Kelch) domain [9]. There are over 40 members of the KLHL family, each representing a unique CRL substrate receptor. KLHL complexes have been shown to ubiquitylate a number of mitotic protein kinases. KLHL9/13 and KLHL21 non-redundantly ubiquitylate Aurora B [10,11], whereas KLHL18 and KLHL22 target Aurora A [12] and PLK1 (Polo-like kinase 1) [13] respectively. KLHL function is also linked to several human cancers. KLHL20 degrades PML (promyelocytic leukaemia protein) and advances prostate cancer progression [14]. Mutations in KLHL37 are associated with brain tumours [15], whereas mutations in KLHL6 are linked to chronic lymphocytic leukaemia [16]. In addition, KLHL12 regulates Wnt signalling by inducing the degradation of dishevelled [17] and also ubiquitylates the COPII (coatomer protein II) component SEC31 [18] as well as the dopamine D_4_ receptor [19].

KLHL proteins have also been implicated in other human diseases. KLHL3 ubiquitylates WNK (with-no-lysine) kinases to regulate ion transport and is mutated in Gordon's hypertension syndrome [20–22]. Mutations are also identified in KLHL7 in retinitis pigmentosa [23], KLHL9 in distal myopathy [24], gigaxonin (KLHL16) in giant axonal neuropathy [25] and KLHL40 in nemaline myopathy [26].

**Figure 1:**
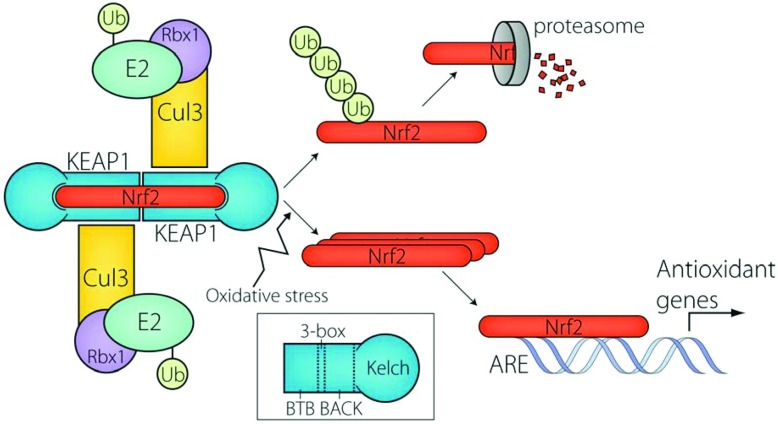
Regulation of Nrf2 Under basal conditions, Nrf2 is polyubiquitylated by the KEAP1–Cul3 E3 ligase and subsequently degraded by the proteasome. Under conditions of oxidative stress, the KEAP1–Nrf2 interaction is destabilized. Nrf2 accumulates and binds to AREs, promoting transcription of cellular defence genes. Ub, ubiquitin.

The best characterized Kelch-like family member is KEAP1 (Kelch-like ECH-associated protein 1) (KLHL19). KEAP1 regulates the oxidative stress response by controlling the levels of the transcription factor Nrf2 (nuclear factor erythroid 2-related factor 2). Under normal conditions, KEAP1 targets Nrf2 for proteasomal degradation [27,28]. Upon cellular stress, KEAP1 oxidation allows Nrf2 release and the subsequent activation of cellular defence genes carrying an ARE (antioxidant-response element) in their promoter [29] (Figure 1).

## Structure of the CRL3s

The typically dimeric BTB domain mediates assembly with two Cul3 subunits leading to a dimeric CRL3 complex. Structural studies on the MATH–BTB protein SPOP (speckle-type POZ protein) identified a further two-helix extension of the BTB C-terminus that was critical for high-affinity Cul3 interaction. Since this motif has a role analogous to that of the F-box and SOCS (suppressor of cytokine signalling) box of other Cullin adaptors, it was termed the ‘3-box’ and appears to be conserved in all BTB adaptor proteins [30]. Subsequent structural characterization of the KLHL family revealed that the 3-box comprises the first two helices of the exclusively helical BACK domain [31,32]. In KEAP1, the connected Kelch domain forms the substrate-binding surface for Nrf2. The Kelch domain is characterized by six Kelch repeat motifs, which form the ‘blades’ of a variable β-propeller domain.

The Cul3 N-terminal domain forms an extended stalk-like structure consisting of three Cullin repeats. In the first repeat, helices 2 and 5 bind the BTB domain similarly to the assemblies of Cul1 and Cul5 with Skp1 and Elongin C respectively [31,32]. A further nine residues at the N-terminus of Cul3 were found to be critical for high-affinity Cul3 assembly and were located in the KLHL11–Cul3 structure in a hydrophobic groove formed by the 3-box [31]. The crystal structure of the C-terminal domain of Cul3 is yet to be determined.

**Figure 2:**
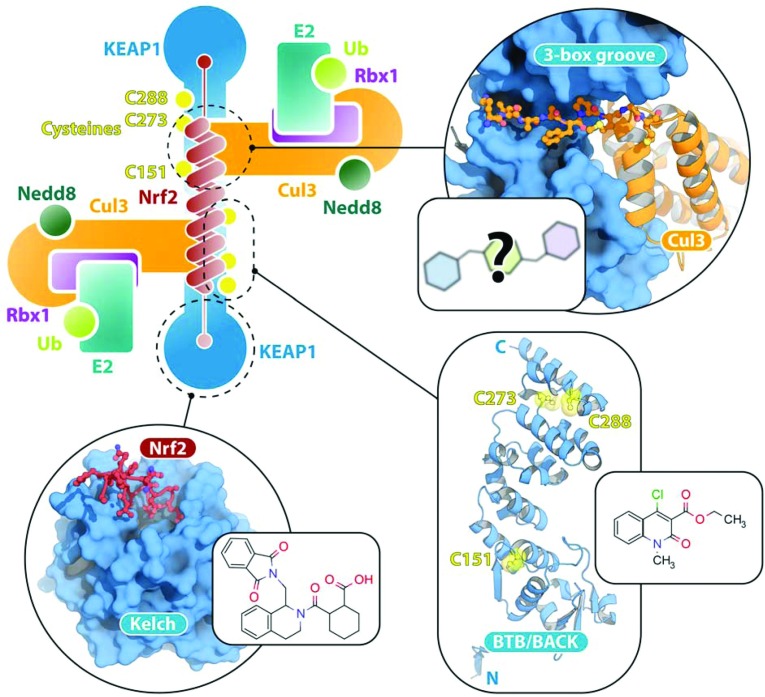
Model of the KEAP1 CRL3 structure and potential sites for small-molecule inhibitors The KEAP1–Nrf2 crystal structure (PDB code 2FLU) [45] is shown alongside an early inhibitor of this interaction [46]. The BTB–BACK domains of KEAP1 are modelled from the structure of KLHL11 (PDB code 3I3N) [31], highlighting the cysteine residues known to be covalently modified by the small molecule displayed [39]. The 3-box groove is also modelled from the KLHL11–Cul3 structure (PDB code 4AP2) [31] showing the Cul3 interface as a potentially druggable site.

The dimeric assembly of the KEAP1 CRL3 complex is essential for the regulation of Nrf2, which contains two degrons, a high-affinity [ETGE (Glu-Thr-Gly-Glu)] motif and a low-affinity [DTG (Asp-Thr-Gly)] motif, separated by a central lysine-rich α-helix [33–36]. Crystal structure and EM reconstruction data indicate that the dimeric CRL3 complex would assemble such that the two substrate-binding Kelch domains are separated by 80–95 Å (1 Å=0.1 nm), an appropriate distance to engage both degrons either side of the central helix [31,34]. This supports the posited ‘two-site binding’ mechanism of the KEAP1–Nrf2 interaction and would potentially leave the central helix exposed to ubiquitylation by two activated E2 enzymes [31] (Figure 2). Geometric comparisons between the Cul3 complexes of KLHL3, KLHL11 and SPOP suggest that the precise spacing between the E2s and the bound substrate may vary in each complex [37]. Additionally, SPOP CRL3 complexes have been shown to form higher-order oligomers that enhance the ubiquitylation activity of the E3 [32].

## CRL3s as drug targets

The ubiquitin system is widely considered to contain a raft of potential new drug targets. Although a challenging prospect, there are early examples of success, such as the proteasome inhibitor bortezomib, now marketed as Velcade® for the treatment of multiple myeloma, and several E3 ligase inhibitors have entered clinical trials, including inhibitors of Mdm2 (murine double minute 2) [2]. KEAP1 is a notable target of interest, as induction of Nrf2 and the antioxidant/anti-toxification response would make an potentially attractive therapeutic strategy in neurodegenerative, cardiovascular, metabolic and inflammatory diseases [38]. The most advanced small molecules covalently modify the reactive cysteine residues within KEAP1 to destabilize its interactions with Cul3 and Nrf2 [39,40] (Figure 2). However, the Phase III clinical trial of bardoxolone methyl was recently abandoned, indicating that this therapeutic strategy is non-trivial [38].

Next-generation small-molecule inhibitors are now being developed to target the Kelch domain of KEAP1 [41–43]. These inhibitors occupy the binding pocket to block directly the interaction with Nrf2 (Figure 2). Ubiquitylation is thereby blocked, allowing cellular Nrf2 levels to accumulate. As proof-of-concept, peptide inhibitors targeted to the same interface have achieved high potency [44]. A further strategy yet to be explored is to target the KEAP1-binding interface with Cul3. For example, molecules could be designed to block the hydrophobic groove of the 3-box, a region known to be important for high-affinity Cul3 interaction [31] (Figure 2). Another possibility is the targeting of the E2–E3 interaction, although a weak binding affinity makes structural study of this interface challenging [2].

The identification of druggable sites has been a major bottleneck for drug discovery in the ubiquitin–proteasome system where many protein–protein interfaces are large and flat. By understanding the structural biology of the CRL3s (and other E3 ligases), it is hoped that possible novel sites and modes of action may be discovered. Additionally, lessons from the KEAP1 pathway may be applied to other Cul3 substrate adapters as our understanding of their functional targets and disease links deepens.
